# Editorial: Nutrigenomics in Animal Health and Production

**DOI:** 10.3389/fgene.2018.00201

**Published:** 2018-06-08

**Authors:** Sami Dridi

**Affiliations:** Center of Excellence for Poultry Science, University of Arkansas, Fayetteville, AR, United States

**Keywords:** nutrigenomics, transcriptomics, metabolomics, stress, heifers, chicken, rodents

Livestock production is facing substantial challenges from steep projected increase in global demand for food and high quality of animal proteins, and the need to adapt to environmental obstacles such as heat and nutritional stress.

There has been a prodigious increase in omics sciences, including nutrigenomics research (Sales et al., 2014), which aimed to understanding the molecular interaction between nutrition, animal genome, and performance under both normal and challenging conditions, and thereby identifying key molecular signatures for subsequent development of effective nutritional strategies to improve feed efficiency, animal well-being as well as livestock production sustainability.

By inviting experts in their fields and gathering reviews, case reports, and original research articles, the objective of the present research topic was to highlight the current progress in animal nutrigenomics which contribute to solving the intervening puzzle between nutrients, genes, and animal performance. Five elegant papers covering wide range of nutritional omics; from nutritional epi-transcriptomics to nutritional metabolomics, in different species (rodents, large mammals, and monogastric) were accepted.

A review by Murdoch et al. discussed the establishment and re-programming of the epigenome and its regulation by nutritional status and nutrient composition as well as the effects of epigenetics changes on livestock production. As an example, Kang et al. (2017) have conducted studies where they imposed psychological (acute and chronic immobilization) and early-life nutritional (food-deprivation) stress on birds and have demonstrated the modulation of key regulators of DNA methylation.

In an original research article, Rajaei-Sharifabadi et al. have shown that *Morinda Citrifolia* (Noni)-rich diet alleviated stress induced by heat exposure in broiler (meat-type) chickens and this effect was found to be mediated through modulation of hypothalamic orexin-AMPK-mTOR pathways.

In attempt to assess the safety of genetically modified (GMO) feed, Sharbati et al. investigated the effect of GMO-maize on rat tissues. By using transcriptome based profiling, Sharbati et al. did not observe, in their research article, any significant GMO maize-related changes in intestinal expression of apoptosis-, DNA damage-, and UPR-related genes.

The final original research article by Golder et al. (2018) aimed to determine the link between nutrients (additives), bovine (dairy Holstein heifer) genotype, and the enteric biome in acidosis challenging conditions. Several putative QTL were identified for various metabolites such as lactate and butyrate, acidosis, and host-microbiome interaction.

In summary, the articles within the current research topic, along with many other publications elsewhere, approve the ability of nutrition-environment interaction in modulating the animal (epi)genome and subsequently its growth, health, and well-being. Despite their descriptive nature due to lack of mechanistic and functional studies, these papers suggested that omics sciences have the potential to change the future of dietary guidelines by identifying key molecular markers for personalized nutrition approaches.

## Author contributions

The author confirms being the sole contributor of this work and approved it for publication.

### Conflict of interest statement

The author declares that the research was conducted in the absence of any commercial or financial relationships that could be construed as a potential conflict of interest.
